# Stressors, emotions, and social support systems among respiratory nurses during the Omicron outbreak in China: a qualitative study

**DOI:** 10.1186/s12912-024-01856-6

**Published:** 2024-03-21

**Authors:** Wenzhen Yu, Ying Zhang, Yunyan Xianyu, Dan Cheng

**Affiliations:** Department of Respiratory and Critical Care Medicine, Renmin Hospital of Wuhan University, No. 238, Jiefang Road, 430060 Wuhan, China

**Keywords:** COVID-19, Working experience, Nurses, Pulmonary medicine, Qualitative research

## Abstract

**Background:**

Respiratory nurses faced tremendous challenges when the Omicron variant spread rapidly in China from late 2022 to early 2023. An in-depth understanding of respiratory nurses’ experiences during challenging times can help to develop better management and support strategies. The present study was conducted to explore and describe the work experiences of nurses working in the Department of Pulmonary and Critical Care Medicine (PCCM) during the Omicron outbreak in China.

**Methods:**

This study utilized a descriptive phenomenological method. Between January 9 and 22, 2023, semistructured and individual in-depth interviews were conducted with 11 respiratory nurses at a tertiary hospital in Wuhan, Hubei Province. A purposive sampling method was used to select the participants, and the sample size was determined based on data saturation. The data analysis was carried out using Colaizzi’s method.

**Results:**

Three themes with ten subthemes emerged: (a) multiple stressors (intense workload due to high variability in COVID patients; worry about not having enough ability and energy to care for critically ill patients; fighting for anxious clients, colleagues, and selves); (b) mixed emotions (feelings of loss and responsibility; feelings of frustration and achievement; feelings of nervousness and security); and (c) a perceived social support system (team cohesion; family support; head nurse leadership; and the impact of social media).

**Conclusion:**

Nursing managers should be attentive to frontline nurses’ needs and occupational stress during novel coronavirus disease 2019 (COVID-19) outbreaks. Management should strengthen psychological and social support systems, optimize nursing leadership styles, and proactively consider the application of artificial intelligence (AI) technologies and products in clinical care to improve the ability of nurses to effectively respond to future public health crises.

**Supplementary Information:**

The online version contains supplementary material available at 10.1186/s12912-024-01856-6.

## Introduction

As of January 29, 2023, more than 753 million confirmed cases of COVID-19 have been reported globally, with more than 6.8 million deaths [1]. The Omicron variant (Omicron, B.1.1.529) is one of the five World Health Organization (WHO) variants of concern (VOCs). Compared with other VOCs, the Omicron variant has significantly increased transmission and immune escape [2]. An analysis by the Chinese Center for Disease Control and Prevention (CCDC) revealed that from December 1, 2022, to early January 2023, Omicron BA.5.2 and BF.7 were the prevalent strains in China, with these two lineages accounting for 97.5% of all indigenous cases [3]. At the press conference of the Joint COVID-19 Prevention and Control Mechanism of the State Council on January 14, 2023, the number of hospitalizations due to COVID-19 reached a peak of 1.63 million on January 5, and from December 8, 2022, to January 12, 2023, a total of 59,938 deaths related to hospitalizations due to COVID-19 occurred in medical institutions across the country. There were 5,503 deaths due to respiratory failure [4].

Nurses play a vital role in rescuing and treating COVID-19 patients. Nurses are at the forefront of the fight against disease, facing enormous physical and mental pressure while adopting effective strategies to overcome unprecedented challenges [5–6]. Research has shown that frontline nurses faced numerous challenges during the COVID-19 pandemic. A systematic review and meta-analysis exploring the impact of the COVID-19 pandemic on the prevalence of psychological symptoms among nurses showed that the pooled prevalence of anxiety, depression, and sleep disturbance was 37%, 35% and 43%, respectively [7]. During the COVID-19 pandemic, the workload of frontline nurses also increased significantly due to multiple factors, such as increased patient requirements and work content, longer work hours, and a shortage of staff and personal protective equipment [8–9]. In addition, nurses also expressed feelings of helplessness and inadequacy because, despite hard work, they were unable to provide dignified and acceptable-quality care [10]. Therefore, it is necessary to emphasize the significance of support for nurses from governments, policy-makers, and nursing organizations to reduce the negative impacts on nurses’ well-being during and after a pandemic or epidemic [11]. Otherwise, nurses may feel burnout, leading to turnover [12–13].

Nevertheless, existing studies on frontline nurses’ work experiences have been conducted predominantly in the context of nurses as physically healthy individuals providing health care services to COVID-19 patients. With the rapid spread of the Omicron BA.5.2 and BF.7 variants, it was estimated that most of the Chinese population was infected in December 2023 [14]. It has been reported that the number of clinic visits due to fever in China peaked on December 23, 2023. Two weeks later, the number of critical hospitalizations for COVID-19 also peaked [4]. During that period, the challenges faced by nurses in China were unprecedented and vastly different from those of other nurses worldwide. Most nurses were both health care providers and infected patients. The present qualitative study aimed to explore the work experiences of frontline respiratory nurses during the Omicron epidemic, develop better nursing countermeasures and management strategies for managers and promote better support for frontline nurses to provide patients with higher-quality care in possible future outbreaks.

## Methods

### Study design

The present study adopted a qualitative descriptive phenomenological design to conduct in-depth interviews. This design is suitable for providing detailed descriptions of participants’ emotions, opinions, and experiences and interpreting the meaning of their behaviours [15].

### Participants and setting

All participants were recruited from a tertiary hospital in Wuhan, Hubei Province, China. A purposive sampling method was used in the present study. To obtain a wide range of experiences, we considered a diverse range of personal details, including age, sex, education level, marital status, years of nursing experience, professional title, type of employment, and workplace type, during the selection of participants. The sample size was determined based on data saturation [16].

The inclusion criteria were registered nurses working at the PCCM who provided direct care to COVID-19 patients between December 8, 2022, and January 8, 2023, and those who expressed willingness to participate in the study and share their experience. Nurse managers and nurses working less than two weeks during the abovementioned period were excluded.

### Data collection

The data were collected through individual and face-to-face, in-depth interviews from January 9 to 22, 2023.

After a literature review and panel discussion, an interview guide was developed. Two pilot interviews were also conducted to investigate the appropriateness of the interview questions, and the guide remained the same. The data from the pilot interviews were not included in the analysis. All interviews were conducted by one researcher (first author), who completed a thorough and systematic study of qualitative research methods and reviewing skills before the start of the study. The final semistructured interview guide consisted of nine open-ended questions (see Supplementary file 1).

The interviewer and the participants had been colleagues for 3–7 years and trusted each other. The interviewer informed the participants about the purpose, voluntariness, anonymity, and confidentiality of the study one day before the interview and scheduled the time of the interview. Interviews were usually conducted on an afternoon when the participants were off duty, or an alternative time was arranged if the participants could not leave work on time. The interviews were conducted in a one-room office to ensure that the environment was quiet and undisturbed so that the participants could express their inner feelings to the interviewer with an open mind. With the participants’ permission, all interviews were audio-recorded using a digital voice recorder. The duration of the interviews varied between 30 and 60 min. Within 24 h of each interview, the audio-recorded data were fully transcribed, and two researchers independently evaluated the data saturation. Any disagreements were resolved through a panel discussion. Behavioural data (laughing, crying, sighing, silence or pausing, etc.) were also recorded during transcription for data analysis. Data saturation was reached at the 10th interview, but an additional interview was also conducted to ensure that no new information emerged. Therefore, a total of 11 respiratory nurses were recruited. None of the nurses dropped out of the study.

### Data analysis

Colaizzi’s method was used to analyse the data [17]. This method involved the following steps: (a) Familiarization: rereading the transcripts verbatim multiple times to become familiar with the data; (b) Identifying significant statements: identifying and extracting meaningful statements relevant to the phenomenon; (c) Formulating meanings: formulating and encoding meanings from important statements; (d) Clustering themes: aggregating the encoded meanings into preliminary themes; (e) Developing an exhaustive description: providing a detailed description of each of the themes generated in step d with the addition of participants’ original statements; (f) Producing the fundamental structure: generating themes to reveal the basic structure of the phenomenon using short and condensed phrases; and (g) Verifying the fundamental structure: presenting the transcripts of the interviews, codes, and themes to the participants for feedback on whether their experience of the phenomenon had been accurately represented. Two independent researchers analysed the data simultaneously.

### Rigor

In this study, Lincoln and Guba’s criteria of credibility, transferability, dependability, and confirmability were utilized to ensure rigor [18]. The following strategies were implemented to achieve credible study findings: conducting semistructured, in-depth interviews with open-ended questions and field notes; transcribing audio-recorded data word-for-word and independently analysing the raw data by two researchers; and asking participants to provide feedback on the transcripts, codes, and themes. Transferability was established by considering maximum variations in participant characteristics and presenting appropriate participant quotes. To facilitate dependability and confirmability, several meetings were held among the researchers to discuss and identify codes, subthemes, and themes.

### Ethical considerations

This study was approved by the research and ethics committees of Renmin Hospital of Wuhan University (Approval NO: WDRY2023-K031). Before the interviews, the details of the study, the expected risks and benefits, and the right to withdraw at any time was verbally explained to all participants, and written informed consent was obtained. After the interviews were transcribed, the participants’ names were deleted instead of their identities (A‒K). To ensure confidentiality and privacy, the text data were stored in a locked cabinet, and the audio data were stored on a password-protected computer.

## Results

### Participant characteristics

A total of 11 nurses, including 10 females (90.9%) and 1 male (9.1%), were included. The mean age was 32.09 ± 5.45 years (range = 24–43 years), and the mean number of years of nursing experience was 10.36 ± 5.50 years (range = 3–21 years). The sociodemographic data are displayed in Table 1.

**Table 1:** Characteristics of participants (total number = 11)

Participant Code	Age (year)	Sex	Educational Level	Marital Status	Professional Title	Years in Nursing	Type of Workplace	Type of Employment
A	34	Female	Bachelor’s degree	Married	Intermediate	12	General ward	contract
B	34	Female	Bachelor’s degree	Married	Intermediate	12	General ward	permanent
C	30	Female	Bachelor’s degree	Married	Intermediate	9	RICU	permanent
D	24	Female	Bachelor’s degree	Single	Primary	3	General ward	contract
E	27	Female	Bachelor’s degree	Married	Intermediate	5	General ward	permanent
F	29	Male	Master’s degree	Single	Intermediate	7	RICU	contract
G	29	Female	Bachelor’s degree	Married	Primary	7	RICU	contract
H	39	Female	Bachelor’s degree	Married	Intermediate	18	General ward	contract
I	43	Female	Bachelor’s degree	Married	Intermediate	21	General ward	permanent
J	30	Female	Bachelor’s degree	Married	Primary	7	RICU	contract
K	34	Female	Bachelor’s degree	Married	Intermediate	13	General ward	contract

Abbreviations: RICU, respiratory intensive care uni

### Thematic results

**Fig. 1:**
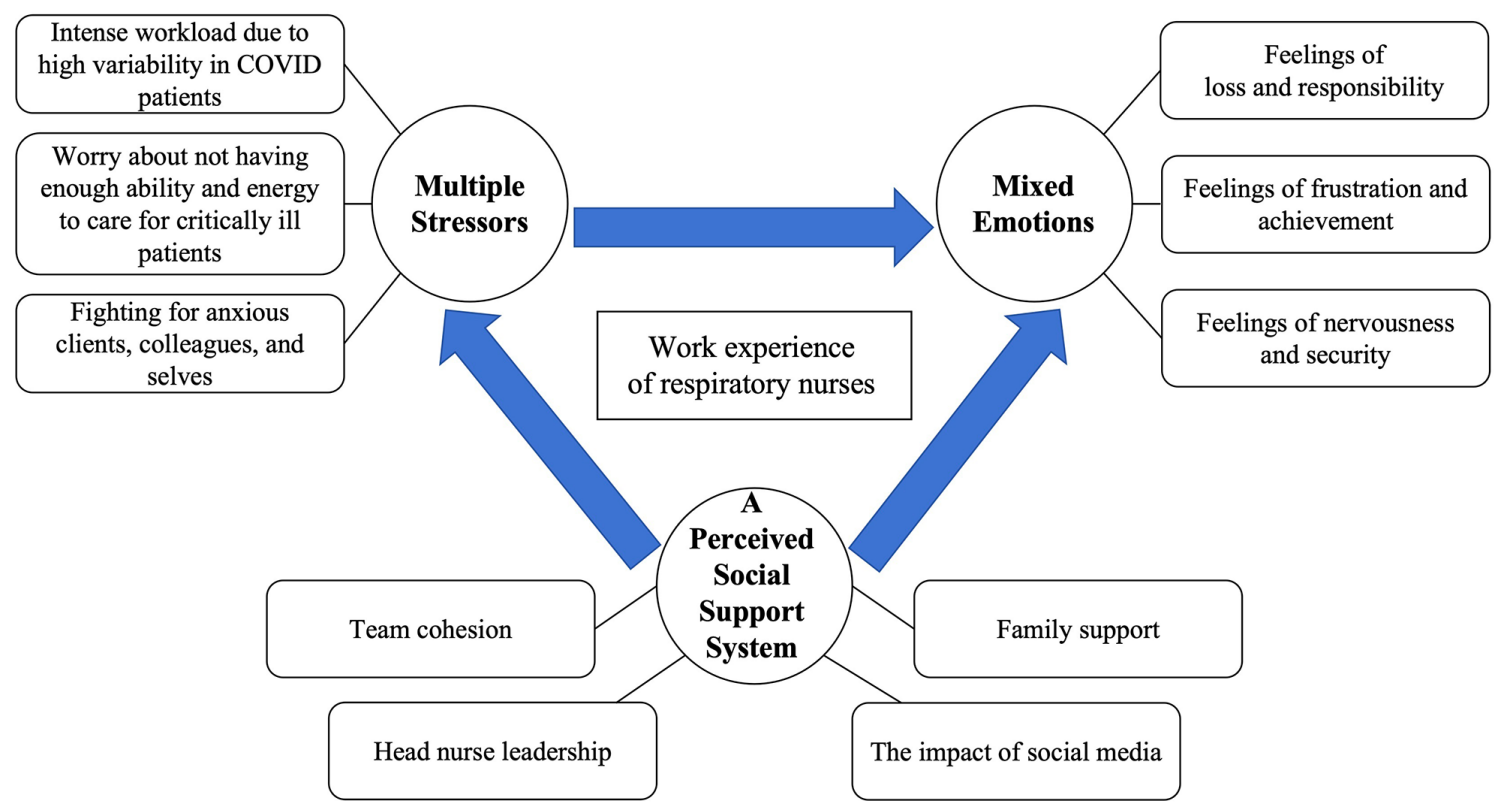
Themes and sub-themes of work experience for respiratory nurses during Omicron outbreak

Three major themes emerged: multiple stressors, mixed emotions, and a perceived social support system. Ten subthemes were identified. The findings are described in Fig. 1.

### Theme 1: Multiple Stressors

 This theme focused on the workplace stressors experienced by respiratory nurses during the Omicron outbreak. Three subthemes were identified in this theme: intense workload due to high variability in COVID patients; worry about not having enough ability and energy to care for critically ill patients; and fighting for anxious clients, colleagues, and selves.

#### Intense workload due to high variability in COVID patients

Most participants reported a high level of work pressure, such as a high number of admissions, a high percentage of critical patients, rapid changes in patient conditions, and frequent resuscitations. As one participant said,

*“For some time now, the RICU has been particularly busy. Every shift is filled with resuscitation cases and the admission of new critically ill patients, usually those who need to be intubated. We borrowed much equipment from the Equipment Division, such as ventilators and high-flow nasal cannula oxygen therapy devices. We usually have enough equipment in our Department, but now we do not.”* (Participant G)

Almost all participants stated that the workload of nursing care associated with COVID-19 had significantly increased, and nurses often had to work overtime to complete their work. As two participants said,

*“Almost all newly admitted patients are given nebulizers and oxygen and undergo urgent arterial blood gas analysis. I could not leave work on time almost daily (bitter smile).”* (Participant C)

*“There are many patients on oral corticosteroids, which is different than usual. I have to talk to the patients about the use and the dosage, tell them when to taper, and talk to the doctor before I give the medication. It all takes time.”* (Participant I)

Another participant said the following:

*“Except for nursing records, I get things done during working hours. Then, I spend off-duty time writing the records.”* (Participant E)

Some participants reported working at an accelerated pace during the work period. One of the participants described their experience as follows:

*“Patients ask me questions, and maybe I am fast in my speech and, well, fast enough in my steps.”* (Participant D)

Most participants reported returning to work after taking a short break from their infections. However, they were still symptomatic when they returned to work. One participant said the following:

*“I had three days of rest and came back to work when my fever was down, and my cough has not gone away yet.”* (Participant A)

#### Worry about not having enough ability and energy to care for critically ill patients

Some of the participants in this study reported significant psychological distress from worrying about not having enough ability and energy to care for critically ill patients. The following excerpts illustrate this subtheme:

*“There are many patients on invasive mechanical ventilation, and the biggest worry is accidental extubation. It is nerve-wracking.”* (Participant F)

*“Some patients are ventilated in the prone position; some are intubated, and some are not. Although the therapeutic efficacy was quite good, at least four colleagues were needed to change the position. It is a big risk at night when we are short-staffed, especially in a resuscitation situation.”* (Participant G)

*“I was worried about making mistakes. During that time, I had night sweats, did not sleep well, often felt weak and dizzy during the day, and was afraid that I would make a mistake while providing care because of my lack of concentration.”* (Participant K)

#### Fighting for anxious clients, colleagues, and selves

In this study, most participants said that patients and their family members, doctors, other nurses, and themselves were experiencing negative emotions such as anxiety. Some participants expressed this as follows:

*“In my communication with patients, I have noticed that many patients are anxious, so I do more explaining than before when I give patients medication. Many patients ask me if their disease is serious…”* (Participant I)

*“Some patients are transferred to the RICU when their condition deteriorates, and their families have no sight of them and are very anxious every day. There is also much pressure on the doctors.”* (Participant G)

*“For us young nurses who are faced with so many critically ill patients who experience rapid changes in their conditions, we often have to communicate with doctors, especially senior doctors. If (we are) inexperienced, communication is slightly difficult. Additionally, because everyone has been working for a long time, it is difficult to know whether (the staff) are irritated or can communicate well with their colleagues. Because after a long shift, they may all be experiencing negative emotions.”* (Participant F)

*“I am not sure if it is because of my illness or because of my work. I often dream about saving patients, probably for both reasons… I hope the hospital will open a free psychiatric and sleep disorder clinic for us.”* (Participant K)

Some participants mentioned maintaining a positive mindset through self-regulation and psychological suggestions as a stress management strategy and expressed the hope that managers would pay attention to the psychological states of frontline nurses and provide psychological support. One participant said,

*“It is important to keep thinking positively. We are all in the same boat now (laughs). The other thing is to learn some relaxation techniques. Leaders should be aware of the psychological dynamics of nurses on the front line and provide psychological comfort.”* (Participant F)

### Theme 2: Mixed emotions

This theme focused on mixed emotional states, that is, the co-occurrence of positive and negative emotions in respiratory nurses during the Omicron outbreak. Within this theme, three subthemes were identified: feelings of loss and responsibility, feelings of frustration and achievement, and feelings of nervousness and security.

#### Feelings of loss and responsibility

 Some of the participants in this study expressed a certain sense of loss. This feeling stemmed from nurses caring for patients, uncertain about when they might become infected, and their lack of a role in taking care of family. One of the participants said,

*“There could still be a psychological setback. I went through the 2020 pandemic in Wuhan, and then I went to another city (to offer support) and witnessed another outbreak. Previously, we thought about how to protect ourselves while helping others. This time, it is unclear how to protect ourselves while treating others.”* (Participant H)

Another participant said,

*“My family members were infected. I was working hard and very busy, and I did not have the extra time or energy to care for them. My parents did not live with me, and I wanted to have time to get them some medicine and check on them. During that time, I was worried about their health because the risks for older people were high. I was worried that their health conditions would become more serious, and I was not caring for them.”* (Participant I)

The majority of the participants in this study stated that they stayed in their jobs despite experiencing substantial and multiple pressures because of a sense of responsibility. One participant, who was asymptomatic and not sure if he was infected, said the following:

*“I think we have to work and stick to the job. First, we have to go to work according to the schedule, which is the most important point, the duty. I cannot stay away from work just because I haven’t been infected. At this most critical point, running away at the first sign of difficulty is impossible. That is certainly not the right thing to do. The main thing is duty because that is one of the most fundamental qualities of an employee.”* (Participant F)

Some participants who had symptoms indicated that their intention in returning to work without fully recovering was to allow other nurses to also have breaks. One participant mentioned,

*“At the time, I had been off for 3 days. Some of my colleagues were just showing symptoms and had no breaks. I thought I should go to work so those colleagues could have breaks, so I picked myself up and came to work.”* (Participant A)

#### Feelings of frustration and achievement

Some of the participants in this study reported that patient blaming made them feel frustrated. Some participants claimed that their frustration stemmed from not seeing a significant improvement in patient outcomes in the short term. Participants described their experiences as follows:

*“When I came back to work after being sick, I had not fully recovered, and occasionally I moved a little slower. Some patients did not understand my situation. I felt despondent at that moment (tears).”* (Participant A)

*“It is very depressing. Intubated patients are difficult to wean from mechanical ventilation for an extended period, and even less severe patients still have symptoms.”* (Participant G)

Most of the participants in this study reported feeling a sense of achievement. The reasons included receiving affirmation from patients or their families; noticing gradual improvement in patient conditions; being helpful to families, friends, or colleagues; and enhancing professional competence. The participants described their experiences as follows:

*“Many patients expressed admiration for my hard work and understood the challenges I faced, some even telling me to take a break. Their empathy motivated me to continue making contributions.”* (Participant D)

*“When the patients were admitted, they were extremely unwell, struggling with speech and reluctant to move. Following treatment, they could eat independently, move about independently, and express gratitude for feeling better. Moments like this bring great happiness to me!”* (Participant H)

*“During this period, I received more calls from acquaintances for counselling and felt fulfilled. They asked questions, such as if azvudine was effective, and I could advise them on the optimal stage for taking medication. Consequently, I felt that I was valued and was motivated to be a respiratory nurse. We are also confident that the mortality rate in our ward is very low, and many patients have been discharged.”* (Participant I)

*“This experience can be considered a form of training, helping us develop specialized skills and gain personal insights. If we face a similar emergency in the future, we will possess greater knowledge and skills regarding how to tackle it.”* (Participant F)

#### Feelings of nervousness and security

Some of the participants in this study expressed nervousness due to the fear of being infected and of passing the virus on to their family members. One participant who tested negative for SARS-CoV-2 antibodies described her feelings as follows:

*“My workmates falling ill affected me. I did not know what the symptoms would be if I got it. It was that uncertainty. Therefore, going to work caused anxiety at the beginning of the outbreak. It is that feeling of not knowing if you will go down next… It is like there’s no escape.”* (Participant H)

Another participant stated the following:

*“I am feeling nervous. I am in daily contact with patients who have tested positive, and since I have elderly relatives and young children at home, I am more concerned about bringing the virus back with me. That is why, when I return home from work, I leave my clothes and shoes outside, and the first thing I do upon entering my home is shower. When I returned home, my children used to hug me, but I would say, “Stay back, stay back.” I had to take a shower before I embraced them. Will there be a second or third wave? Can elderly people and children withstand this? Will my health worsen over time?”* (Participant B)

Some of the participants expressed that their work in the PCCM made them feel reassured:

*“I feel that working in a hospital makes it easier to get help if I become infected. As a respiratory staff member, I feel safe.”* (Participant K)

“*It is not really that worrying. I think I was in the PCCM, and if anything happened to me, everyone would save me. I’m in this department, and the backup is strong.*” (Participant C)

### Theme 3: Perceived social support systems

The vast majority of participants talked about the social support systems they perceived and how these social support systems impacted them. Within this theme, four subthemes were identified: team cohesion, family support, head nurse leadership, and the impact of social media

#### Team cohesion

Most participants in this study reported that coworkers helped each other at work, comforted each other psychologically, and were more unified than before the epidemic. The following descriptions represented this subtheme:

*“During that time, even though almost everyone was sick and very busy at work, the atmosphere in our department was amiable. Every time you were busy, others would come to help you, and so would I. No one slacked off or hid from work, and everyone worked hard. It was a positive boost because no one was dragging their feet.”* (Participant B)

*“In such a busy situation, our colleagues are more united. We help each other. It is more cohesive. Busier, but more in touch (smile).”* (Participant C)

*“After my colleagues got infected, they shared some of their feelings with me. It was not really that uncomfortable, so my mind quickly relaxed. When people’s symptoms subsided, their temperature dropped, or the pain in their bodies eased, you could sense their happiness. I also felt happy when I heard such news. I feel that this kind of happiness is different from usual.”* (Participant H)

#### Family support

 Some participants in this study indicated that the health and support of their families strongly supported them in focusing on fighting against the outbreak:

*“My family was very supportive (laughs). Everyone was very supportive. They were trying to minimize my burden. Because I did not know if I was infected, but when they were infected, they drank water, took their own medicine, and took their temperature. They wore masks, and they disinfected at home. I think that this was also a kind of support. They did not delay buying food or cooking every day and did not stop cooking or eating just because they were lethargic after the infection. Therefore, I think that is a kind of support (laughs).”* (Participant H)

*“I think my family… my support system is stable (grin), so I think I would be fine (to work).”* (Participant C)

#### Head nurse leadership

Some of the participants in this study indicated that the head nurses’ leadership had a significant impact on the nurses’ work experiences:

*“Rational scheduling and decision-making by the nurse managers is important. Pairing senior nurses with junior nurses during scheduling can avoid several risks. It is also important to try to ensure that everyone gets enough rest while maximizing the potential of the frontline nurses.”* (Participant F)

*“One day, the on-call shift started. Zhang was on it, and she did not get a moment’s rest until the end of the shift, and neither did we. She came to help us. She helped everyone. Where we were busy, where she was, arranging that shift helped our whole team and individuals a lot.”* (Participant B)

*“Any shortage of supplies or equipment or emergency, just talk to the head nurse, and it all gets resolved, so it is not so draining to work.”* (Participant D)

#### Impact of social media

In this study, some participants mentioned that social media use impacted their psychological feelings, as follows:

*“There are some very positive short videos online. One of our colleagues and some well-known people have shared their personal experiences fighting the outbreak, and it has been helpful to see others actively confronting it.”* (Participant H)

Some participants expressed the opposite view:

*“It worries me a little bit because the reinfections that are rumoured online can be scary.”* (Participant C).

## Discussion

This study describes the challenges faced by respiratory nurses caring for COVID-19 patients during the Omicron outbreak in China from late 2022 to early 2023. Specifically, the findings interpreted these experiences as multiple stressors, mixed emotions, and perceived social support systems.

Like in the study by Al Maqbali M [7], a significant proportion of participants in our study reported that they had psychological problems such as stress, anxiety, frustration, or sleep disturbance and expressed a need for psychological support. Falatah’s [12] study showed that nurses’ turnover intentions increased significantly during the COVID-19 pandemic compared with that before the pandemic, and stress, anxiety, and fear of disease were predictors of nurses’ turnover intentions. In contrast to those in other studies, the participants in our study expressed their sense of security, which stemmed from confidence in their own professional background and trust in their colleagues. A previous study emphasized that understanding the psychological needs of frontline nurses and providing them with tailored psychological support can improve their mental health status and promote quality responses to clinical nursing and public health emergencies [19]. In addition, a cross-sectional correlation study conducted by Hoşgör [20] revealed that there was a significant positive correlation between nurses’ psychological resilience and job performance during the COVID-19 pandemic. These findings show that adopting strategies to improve the psychological resilience of nurses is helpful for optimizing the efficiency of nursing work and improving the quality of patient care. Therefore, during a public health crisis, nurse managers should assess the mental health status of frontline nurses in a timely manner, understand in depth the sources of pressure experienced by nurses, and establish psychological treatment teams to provide offline or online psychological support in the form of one-on-one or group support to improve the mental resilience and physical health of nurses.

In our study, participants described their sources of perceived social support, such as support from their teams, family members, head nurses, and social media. This social support helped them cope with the challenges during this difficult time and encouraged them to provide nursing care to the best of their ability. The participants had positive expressions and emotions when discussing their perceived social support systems. These findings are consistent with the findings of the Shen study [21], which revealed that the greater the level of social support, the better the psychological condition of nurses during the COVID-19 pandemic. Therefore, we strongly recommend that hospital managers regularly visit clinics, interact with frontline nurses, praise their vital role in dealing with the outbreak, and take comprehensive measures to increase value awareness, including compensation, honorary certificates, and publicly recognizing nurses’ contributions. In addition, visiting nurses on the frontline will help address difficulties such as shortages of equipment and human resources in the early stages of outbreaks.

Conversely, some participants in our study reported that rumours on social media about the serious consequences of reinfection negatively affected them. This may be related to the fact that most of the study participants were both patients and caregivers at the beginning of the outbreak. This points to the importance of leading public health experts being organized by the executive branch to provide evidence-based information to the public through social media.

Consistent with the findings of previous research [22], some participants described concerns not only about their own health but also about the health of their family members. This highlights the necessity of extending support for frontline nurses to their family members, including providing medicine and medical counselling. In addition, developing contingency plans to ensure the timeliness and accessibility of social support systems is an issue that managers must address.

The results of this study showed that flexible shift scheduling, active communication, timely resolution of problems, and close working cooperation with nurses played crucial roles in facilitating frontline nurses’ responses to the outbreak. Nursing managers are critical in maximizing the retention of nursing human resources and maintaining productivity and efficiency in health care organizations. Nursing leadership styles strongly influence nurses’ happiness and work environments. Niinihuhta [23] suggested that nurse leaders should use a supportive and relationship-focused leadership style. Another systematic review conducted by Cummings [24] provided robust evidence that relational leadership styles, such as transformational and authentic leadership styles, are significantly associated with improved outcomes, including outcomes regarding job satisfaction, employee-work relationships, employee health and well-being, the organizational environment and productivity.

In contrast, leadership focusing only on task completion is insufficient for achieving positive nursing health and workforce outcomes. As revealed in the scoping review conducted by Sihvola [25], nurse leaders should adopt a relational leadership style and positive communication to support nurse resilience during the COVID-19 pandemic. Furthermore, as an extension of the relational leadership style, inclusive leadership could increase the psychological ownership of nurses and reduce turnover intentions [26].

Unlike in previous situations, most participants in our study had symptoms, such as coughing or weakness, while caring for their clients. Therefore, as the bellwether of frontline nursing caregivers, head nurses should consider the overall situation of hospital management when public health emergencies occur, pay attention to the needs of frontline nurses, consider nurses’ advice, tolerate nurses’ shortcomings and mistakes, and construct an organizational relationship with clear and transparent communication, updated information, flexible shift arrangements, and mutual trust among colleagues to achieve the common goals of organizations and individuals to defeat the pandemic.

According to the results of the present study, respiratory nurses generally work longer hours in the event of an outbreak. At the beginning of the outbreak, the care workload surged as a large number of patients flooded hospitals. As a result, the amount of time to required complete nursing records increased. Consequently, bedside care was commonly provided to patients during normal business hours, and care notes were commonly completed during off hours. In addition, staff shortages were exacerbated by the infection of most logistics staff, and nurses had to take over delivering meals to patients and transporting medical and living supplies.

To alleviate the acute shortage of nursing staff and improve the quality and efficiency of nursing care, attempts are being made worldwide to apply AI technology to care, including COVID-19 care. Kagiyama [27] reported that a telemedicine-based self-vital sign examination system could quickly and accurately obtain vital sign information by measuring and uploading COVID-19 patient data without the risk of spreading infections. Mairittha [28] integrated a spoken conversation system into a smartphone application for care records. They found that the method increased the documentation speed by approximately 58.3% compared to the traditional keyboard-based method. Alderden [29] explored an AI-based transparent machine learning model that could predict the risk of hospital-acquired pressure injuries in ICU patients with COVID-19. Other studies have shown that nurses already use AI to perform various tasks across multiple patient populations, such as assisting elderly patients or recovering patients with exercise and in pain management, communication, interviewing, and patient education [30]. Nurses should recognize the need using AI in care. Nurses should increase their awareness of AI development; actively communicate and collaborate with experts in related fields; and advocate for patient and nurse involvement in the design, implementation, and evaluation of all aspects of AI health technology to prepare for possible future public health events.

### Limitations

All participants in this study were from a tertiary hospital in Wuhan, China. Therefore, the results of the current study may not be generalizable to other settings. Despite we utilized purposive sampling method to ensure diversity of opinions, the majority of participants were female, which was due to the relatively small proportion of male nurse in China. In addition, although our interviews began one month after the start of the outbreak, they took place for two weeks, which may have influenced the views and expressions of the participants over time.

## Conclusion

Respiratory department nurses provided insight into their work experiences during the Omicron outbreak in China from late 2022 to early 2023. Despite experiencing exhaustion, nurses continued to take care of COVID-19 patients with the sense of responsibility of “angels without wings.” Respiratory nurses also experienced a sense of accomplishment from helping patients and a sense of security from their professional backgrounds. The mutual help of team members, support from family members, leadership by head nurses, and influence of social media are essential factors supporting frontline respiratory nurses in the fight against COVID-19. Hospital administrators should pay attention to the pressure and needs of frontline nurses during epidemics, improve psychosocial support systems, optimize the leadership styles of nurse managers, and actively explore the use of AI in the field of clinical nursing to improve nurses’ abilities to respond to public health emergencies.

### Implications

The findings of this study reveal the multiple stressors and mixed emotions encountered by frontline respiratory nurses in combating COVID-19, which is helpful for nurse managers to develop comprehensive strategies that mitigate the adverse impact of these stressors and the negative emotions on nurses’ well-being and augment the positive emotions’ influence on nurses’ work engagement. Moreover, the identification of the nurses perceived social support system would assist policy-makers and hospital administrators in formulating more tailored polices to enhance their support for frontline nurses. Additionally, the design and implementation of training programs focusing on respiratory intensive care for nurses and leadership skills for charge nurse, will play a crucial role in effectively responding to extreme pandemic events. Furthermore, the researchers recommend that more qualitative research be carried out in different medical institutions and that more male nurses be included to improve understanding of the phenomenon. It is also suggested that further research be conducted to explore the psychosocial support needs of frontline nurses and ultimately improve their mental and physical health and quality of care for COVID-19 patients.

### Electronic supplementary material

Below is the link to the electronic supplementary material.

Supplementary Material 1

## Data Availability

The datasets generated and/or analyzed in this study are not publicly available because the data contain individual participant information, but are available from the corresponding author on reasonable request.

## Abbreviations

PCCM: Department of Pulmonary and Critical Care Medicine
COVID-19: Novel coronavirus disease 2019
AI: Artificial intelligence
WHO: World Health Organization
VOC: Variants of concern
CCDC: Chinese Center for Disease Control and Prevention
RICU: Respiratory intensive care unit
