# Supplementary material for: Stressors, emotions, and social support systems among respiratory nurses during the Omicron outbreak in China: a qualitative study
Source: BMC Nurs. 2024 Mar 21;23:188. doi: 10.1186/s12912-024-01856-6 (PMC10956170; doi:10.1186/s12912-024-01856-6)
Supplement: Supplementary file 1 — Supplementary Material 1 [file 12912_2024_1856_MOESM1_ESM.docx]

**Supplementary file 1:** Interview Guide

1. Could you talk about the specifics of caring for patients with COVID-19 between Dec 8, 2022, and Jan 8, 2023?
2. How did you feel about your work during that period?
3. What factors make you feel positive about your work?
4. What factors make you feel negative about your work?
5. What makes you keep working?
6. Was there anything you are worried about?
7. What difficulties did you encounter during your work? How did you solve them?
8. What suggestions do you have for managers?
9. Is there anything else you would like to talk about?
